# Process evaluation of a tailored intervention to Reduce Inappropriate psychotropic Drug use in nursing home residents with dementia

**DOI:** 10.1186/s12877-021-02357-w

**Published:** 2021-07-03

**Authors:** Claudia M. Groot Kormelinck, Charlotte F. van Teunenbroek, Sytse U. Zuidema, Martin Smalbrugge, Debby L. Gerritsen

**Affiliations:** 1 Department of General Practice and Elderly Care Medicine, University of Groningen, University Medical Center Groningen, HPC FA21, P.O. Box 253, 9700 AD Groningen, the Netherlands; 2 Department of medicine for older people, Amsterdam Public Health research institute, Amsterdam UMC - Vrije Universiteit, Amsterdam, the Netherlands; 3 Department of Primary and Community Care, Radboud University Medical Center, Radboud Institute for Health Sciences, Radboudumc Alzheimer Center, Nijmegen, the Netherlands

**Keywords:** Process evaluation, Complex intervention, Participatory action research, Nursing home, Neuropsychiatric symptoms, Psychotropic drugs

## Abstract

**Background:**

Research suggests that collaborative and tailored approaches with external expertise are important to process implementations. We therefore performed a process evaluation of an intervention using participatory action research, tailored information provision, and external coaching to reduce inappropriate psychotropic drug use among nursing home residents with dementia. The process evaluation was conducted alongside a randomized controlled trial assessing the utility of this approach.

**Methods:**

We used Leontjevas’ model of process evaluation to guide data collection and analysis, focusing on the relevance and feasibility, extent of performance, and barriers and facilitators to implementation. Data on the relevance and feasibility and on the extent of performance were collected using a questionnaire targeting internal project leaders at nursing homes and our external coaches. Implementation barriers and facilitators were identified by individual semi-structured interviews. The Consolidated Framework for Implementation Research was used to structure and describe the identified barriers and facilitators.

**Results:**

The intervention was viewed positively, but it was also considered time consuming due to the involvement of many people and designing a tailored action and implementation plan was viewed as complex. The extent of performance differed between nursing homes. Delays in implementation and suboptimal execution of actions may have reduced effectiveness of the RID intervention in some nursing homes. Barriers to implementation were reorganizations, staff turnover, communication issues, unclear expectations, and perceived time pressures. Implementation also depended on the involvement and skills of key stakeholders, and organizations’ readiness to change. Although external coaches stimulated implementation, their additional value was rated variably across organizations.

**Conclusions:**

Barriers to implementation occurred on several levels and some barriers appear to be inherent to the nursing home environment and could be points of leverage of future implementation trajectories. This underlines the importance of assessing and supporting organizations in their readiness to change. Sensitivity analyses, taking into account the week in which nursing homes started with implementation and the degree to which actions were implemented as intended, will be appropriate in the effect analyses of the trial.

**Supplementary Information:**

The online version contains supplementary material available at 10.1186/s12877-021-02357-w.

## Background

Neuropsychiatric symptoms (NPS) are common in nursing home (NH) residents with dementia. Over 80% exhibits NPS such as agitation and apathy [1]. NPS are often treated with psychotropic drugs (PDs), including antipsychotics, hypnotics or sedatives, anxiolytics, antidepressants, anticonvulsants, and anti-dementia drugs [2–5]. However, PDs are associated with significant side effects. Antipsychotics may increase the risk of stroke and mortality [2, 6]. They are also associated with extrapyramidal symptoms and drowsiness [7]. The use of sedatives, hypnotics, antidepressants, and benzodiazepines is associated with falls [8]. In addition, there is evidence that PDs are of limited effectiveness on NPS in residents with dementia [9, 10], especially when used in the long-term [11]. Despite these concerns, about 60% of NH residents uses PDs for NPS and only 10% of PDs are prescribed fully appropriate, with indication, evaluation and therapy duration contributing to inappropriate use [12]. Consequently, the appropriateness of prescribing should be optimized. Moreover, clinical guidelines recommend restricted use of PDs and propose non-pharmacological interventions as first-line treatment for managing NPS [13, 14]. As a result, various interventions have been developed over the years, aimed at reducing inappropriate prescribing and/or targeting a greater use of non-pharmacological interventions in practice [15–19]. Often, these interventions comprise complex, multicomponent interventions [20]. However, the strength of the effects varies for complex and multicomponent interventions to reduce inappropriate psychotropic drug prescribing or to increase the use of non-pharmacological interventions among nursing home (NH) residents with dementia. Although complex interventions can be effective [21], to date they have tended to have relatively small or lacking effects [16, 22, 23], with suboptimal implementation emerging as a prime reason. Process evaluations in NHs have demonstrated that suboptimal implementation results from barriers [24–26], not least of which are skepticism about using non-pharmacological approaches [27]. From a broader healthcare perspective, implementation problems relate to perceptions that the issue is not a priority [28], and the use of standardized “one size fits all” solutions [20]. Complex healthcare interventions may work best if tailored to local circumstances rather than being standardized [20, 28], especially if they identify and target modifiable barriers to change before implementation [28, 29]. Consistent with this, process evaluations of complex interventions among NH residents with dementia have underlined that we must adapt to the specific needs and features of each care organization [24, 26]. It also appears that collaborative approaches that introduce external expertise can address the concerns and problems faced by NH staff, while ensuring awareness of their preferences and increasing awareness [30].

This information was taken into account when designing the Reducing Inappropriate Psychotropic Drug Use in NH Residents with Dementia (RID) study [31]. We hypothesized that implementation would be facilitated if NH staff were actively involved in determining the problem(s) and potential solutions, if interventions could be tailored to the local setting, and if the implementation was guided by a coach. To resolve the challenges of existing strategies, we developed a RID intervention that incorporated three active elements [1]: participatory action research (PAR), which allowed staff to formulate problems and potential solutions concerning inappropriate psychotropic drug use (PDU) and neuropsychiatric symptom (NPS) management [2]; tailored information provision about inappropriate PDU and NPS management; and [3] external coaching. It was anticipated that these active elements would lead to the implementation of a tailored action and implementation plan (AIP) to reduce inappropriate PDU for NH residents with dementia [31]. Including a process evaluation can then provide insight into the true contribution of the intervention to daily practice, helping us to understand why the intervention was successful or unsuccessful, and indeed, how it could be optimized [20]. Information on the relevance and feasibility of a given intervention, as well as the extent of performance, is essential for the credibility of research. For example, difficulties with implementation may result in low treatment fidelity, meaning that the intervention could not be carried out as intended, which in turn may lead to a loss of effect [32].

In this research, we aimed to present the process evaluation of the RID study. To our knowledge, no other researchers have evaluated the implementation of such a complex and tailored intervention in NHs. Given its three active elements, we hypothesize that the degree of implementation of the intervention will be good.

## Methods

### Design

Alongside an effect study, executed between July 2016 and December 2018, a process evaluation was conducted between March 2018 and January 2019. The study was performed in sixteen Dutch NHs caring for residents with dementia who reside in Dementia Special Care Units (DSCU). DSCUs designed to deliver care for residents with Korsakov syndrome, acquired brain injury, Down syndrome, or young-onset dementia were excluded. In- and exclusion criteria were not proposed for the internal project leader, although the tasks and aspects associated with this role were communicated with the NHs (such as creating support, logistics management and provision of information). The external coaches needed to be knowledgeable about dementia and have previous consultation expertise in NHs. More information about recruitment and in- and exclusion criteria can be found in our protocol article [31]. The effect study constituted a two-armed, stepped wedge, cluster randomized controlled trial (RCT) [33]. The data collection period was 16 months per NH, split into two 8-month periods. In the first period, eight NHs started with the RID intervention and another eight NHs deferred the intervention. In the second period, the NHs that started with the intervention continued with implementation of the AIP and the other eight NHs started the intervention. Thus, we had an intervention group and a deferred intervention group, with measurements performed at 0, 8, and 16 months. Participation was on a voluntary basis. Besides the external coaching being offered freely to participating NHs, no financial incentives or additional external influences were provided. Further information about the RID study is provided elsewhere [31]. Since information from the process evaluation can be incorporated in the effect analyses [34], we performed the process evaluation before conducting the effect analyses of the RCT.

We used Leontjevas’ model to guide the collection and evaluation of process data [34]. In the process evaluation, we investigated whether our RID intervention with the active elements PAR, tailored information provision, and external coaching successfully addressed the problems identified with earlier studies. Specifically, we studied two elements [1]: the intervention quality, consisting of the relevance and feasibility of the RID intervention and the extent of performance of the RID intervention (degree of implementation); and [2] the barriers and facilitators to implementation.

The RID study was registered with the Netherlands Trial Registry (NTR5872) on May 27, 2016, https://www.trialregister.nl/trial/5719. We complied with the Consolidated Standards of Reporting Trials (CONSORT) guidelines in conducting and reporting this study [35].

### RID intervention

The RID study examined the effectiveness of an intervention to change practice through cooperation between practice and research, using PAR, external coaching, and tailored information provision to implement tailored AIPs to reduce inappropriate PDU. A multidisciplinary project team (MPT) in each NH (including an internal project leader), an external coach, and researchers, were given specific tasks within the cyclic intervention.

1. *Organizing stakeholder efforts:*

- Researchers organized a kick-off meeting in the NH.
- NHs formed an MPT consisting of at least nursing staff, psychologist(s), physician(s), and an internal project leader. The MPT preferably included stakeholders, such as management and representatives of the residents. Each MPT was supported during their intervention period by an external coach.
- Throughout the process, the MPT and external coach had several meetings (total number was not pre-defined).

2. *Problem analysis:*

- Researchers carried out a problem analysis (both quantitative and qualitative data) using interviews and questionnaires. Problems (as perceived by NH staff) regarding inappropriate PDU and NPS management were examined (observation phase).
- Researchers presented the results of the tailored problem analysis to the MPT and external coach, which was followed by interpretation and reflection in the context of the local NH (reflection phase).

3. *Designing tailored AIP*

- The MPT and external coach created an AIP that matched the identified problems (planning phase).
- The external coach and researchers provided feedback on the AIP (relevance and feasibility of actions, concreteness).

4. *Implementation of tailored AIP*

- The MPT started by implementing the tailored AIP (action phase).

5. *Monitoring progression*

- Researchers carried out an interim measurement on inappropriate PDU. The eight MPTs that started as the intervention group were given interim results at 8 months (observation phase).

6. *Stimulating progression*

- The external coach and MPT discussed and reflected on the interim results (reflection phase).

7. *Adjustments to tailored AIP*

- The MPT was able to adjust the AIP based on the interim results (planning phase) and implement any changes during the second period (action phase).

8. *Providing the final results*

- Researchers carried out a final measurement with respect to inappropriate PDU and provided the MPT with their final results after 16 months.

### Intervention quality and barriers and facilitators to implementation

Table 1, supplemented by Additional File 1, provides an overview of the operationalization of the intervention quality and of the barriers and facilitators to implementation. We examined the quality of the RID intervention. For the relevance and feasibility of the RID intervention, the role of each (group of) stakeholder(s) was evaluated (i.e., researchers, internal project leaders, MPTs, and external coaches). For the extent of performance, each intervention task was evaluated with respect to whether implementation was as intended. We then evaluated the barriers and facilitators to implementation. We did not evaluate the relevance and feasibility of the AIPs. Given that the RID intervention focused on PAR, tailored information provision, and external coaching, we decided that evaluating the relevance and feasibility of each of the self-created and highly variable actions would be impractical and offer limited information in the confines of this study. However, we did examine the extent of performance on the AIP tasks because of their expected impact on the overall effectiveness of the RID intervention.

**Table 1:** Indicators and Operationalization of Intervention Quality, including Barriers and Facilitators to Implementation

INTERVENTION QUALITY		
**Relevance and Feasibility of RID Intervention**		
***Stakeholder***	***Indicator***	***Source***
1) Researchers	Added value tailored information provision	Questionnaire: Likert scale
	Experiences with researchers	Interviews: description
2) Internal project leader & MPT	Competence ^A^ of project leader *(perceived by coach)*	Questionnaire: Likert scale
	Experiences with project leaders	Interviews: description
	Experiences with MPT	Interviews: description
3) External coach	Added value of coaching	Questionnaire: Likert scale
	Coaching necessity for (continued) implementation	Questionnaire: Yes/No
	Competence ^A^ of coach (perceived by project leader)	Questionnaire: Likert scale
	Experiences with coaching	Interviews: description
**Extent of Performance of RID Intervention**		
***Task***	***Indicator***	***Source***
1) Organizing efforts of stakeholders		
- Researchers	Kick-off meeting in nursing home	Questionnaire: Yes/No
- MPT	Formation of an MPT	Questionnaire: Yes/No
	Attendance physicians, psychologists, and nursing staff at MPT meetings ^B^	Questionnaire: % attendance ^B^
- External coach	Meetings coach and MPT in nursing home ^C^	Questionnaire: # meetings
	(Phone) meetings coach and project leader ^C^	Questionnaire: # meetings
2) Problem analysis	Researchers carried out problem analysis and presented results to the MPT and coach	Questionnaire: Yes/No
3) Designing tailored AIP	AIP created	Questionnaire: Yes/No
	Contribution coach, project leader, and MPT to designing the AIP	Questionnaire: Likert scale
	Perceived match between problems and actions	Questionnaire: Likert scale
	Coach provided feedback on the AIP	Questionnaire: Yes/No
	Researchers provided feedback on the AIP	Questionnaire: Yes/No
	Adjustments to AIP based on feedback	Questionnaire: Yes/No
4) Implementation of tailored AIP	Start with implementation ^D^	Questionnaire: # weeks passed
	Execution actions as intended: ^E^ Implementation score	Questionnaire: 10-point scale
5) Monitoring progression	Researchers carried out interim measurement and provided the MPT with the results ^*^	Questionnaire: Yes/No
6) Stimulating progression	Coach discussed and reflected on interim results with the MPT ^*^	Questionnaire: Yes/No
7) Adjustments to tailored AIP	MPT adjusted the AIP based on interim results ^F *^	Questionnaire: Yes/No Interviews: description
8) Providing final results	Researchers carried out final measurement and provided the MPT with the end results	Questionnaire: Yes/No
**Barriers and Facilitators to Implementation**		Interviews: data structured with CFIR

*Abbreviations*: *AIP* Action and Implementation Plan, *CFIR* Consolidated Framework for Implementation Research, *MPT* Multidisciplinary Project Team

^A^ Evaluated as: (very) competent on content (PDs, alternatives in managing NPS) and process (motivate, structure)

^B^ Since these disciplines are directly managing PDs and NPS, their attendance was considered most important. For each NH, the % of attendance was given as a mode (most frequently occurring % of separate disciplines). Attendance of separate disciplines is depicted in Additional File 2

^C^ The MPT and coach were supposed to have regular contact, but the number of meetings was not pre-defined

^D^ 8 weeks were planned for the problem analysis and designing the AIP, leaving 6 or 14 months (short vs. long duration) for implementation: Implementation within 8 weeks is as intended, 8–16 weeks suboptimal, > 16 weeks is deviation

^E^ Mean of Implementation scores of each action from AIP: 10-point scale (0 not at all implemented as intended – 10 totally implemented as intended) per action

^F^ Providing MPTs with their interim results was supposed to provide NHs with the opportunity to adapt the AIP. Not making changes while results indicated no improvement with respect to inappropriate PDU is considered a deviation

^*^ Only for the 8 NHs who started in the intervention group

### Data collection

After the final measurement, the researcher (CGK) sent a web-based questionnaire to the internal project leader and external coach at each NH. Approximately 1 or 2 weeks after completion of the questionnaire, the same researcher (CGK) held individual semi-structured telephone interviews with the internal project leader and external coach. The information derived from the questionnaires was explored in-depth during the interviews, to gain a more thorough understanding. The duration of an interview was approximately 1 h. As with the interviews, the questionnaires were completed individually at a time that was convenient for them. External coaches evaluated the process for each NH separately. Relevance and feasibility were examined using both sources, while the extent of performance was based only on the questionnaire. Given that our study was designed to consider the barriers and facilitators identified in previous studies, we explored this matter in depth based on data from the telephone interviews. The telephone interviews were audio recorded and transcribed verbatim.

### Data analysis

Quantitative data were analyzed with IBM SPSS version 25 (IBM Corp., Armonk, NY, USA), using descriptive statistics. Qualitative data were analyzed by deductive content analysis [36], using the Consolidated Framework for Implementation Research (CFIR) to structure and describe the barriers and facilitators [37]. Two researchers independently coded the data (CGK and DG), with another three authors available for discussion in case of disagreement (CVT, MS, SZ). The process evaluation focused on a general evaluation of the implementation process, but we also examined differences in the extent of performance among NHs.

## Results

The respondent characteristics are summarized in Table 2. All 16 internal project leaders participated in the process evaluation, although one completed the questionnaire partially and one did not respond to the request for an interview. All six external coaches also participated in the process evaluation, and a completed digital questionnaire was received for each NH; however, due to a change of jobs, one external coach could not give an interview for one of the NHs. Therefore, we have data for 31 questionnaires and 30 interviews (response rates of 97 and 94%, respectively). The majority of respondents is female (over 80%). The respondents have varying educational backgrounds and current positions (see Table 2).

**Table 2:** Participant Characteristics

Internal project leader (*n* = 16)		External coach (*n* = 6)	
Female, *n* (%)	*14* (88%)	Female, *n* (%)	*5* (83%)
Function, *n* (%)		Education, *n* (%) ^d^	
Elderly care physician	*5* (31%)	Health sciences	*2* (33%)
Nurse ^a,b^	*4* (25%)	Public administration	*1* (17%)
Team leader ^c^	*2* (13%)	Business economics	*1* (17%)
Project employer	*2* (13%)	Health business Administration	*1* (17%)
Policy advisor	*1* (6%)	Social sciences	*1* (17%)
Quality and policy employer	*1* (6%)		
Training coordinator	*1* (6%)		

^a^ Including two specialized gerontology and geriatrics nurses

^b^ One nurse also being the care director

^c^ One team leader also being behavior consultant

^d^ Highest education. In addition, all coaches are experienced in health care, four of which have followed a professional nurse education. All coaches were at the time employed by Vilans as (senior) consultant

### Intervention quality: relevance and feasibility of the RID intervention

The results for the quantitative evaluation of the contributions by researchers, internal project leaders, MPTs, and external coach are summarized in Table 3. The majority of the respondents perceived the tailored information provision to be of high added value. External coaching was perceived to be of high added value by the majority of the internal project leaders, whereas the majority of the external coaches indicated that coaching was of added value ‘to a reasonable extent’. The majority of the external coaches indicated that coaching is a necessity for (continued) implementation, while the majority of the internal project leaders indicated this is not a necessity. Both the majority of the internal project leaders as well as the external coaches perceived the other party to be competent or very competent. The qualitative evaluation is provided below.

1. Researchers

**Table 3:** Relevance and Feasibility of the RID intervention

	Internal project leader *N* = 15 *N,* (%)	External coach *N* = 6 ^a^ *N,* (%)
1) Researchers		
Added value tailored information provision ^b^		
Strongly	*9* (60%)	*12* (75%)
To a reasonable extent	*5* (33%)	*3* (19%)
To some extent	*1* (7%)	*1* (6%)
2) Internal project leader		
Competence of project leader ^c^ perceived by coach		
Competent or very competent	N.A.	*9* (56%)
Not competent/not incompetent	N.A.	*3* (19%)
Other ^d^	N.A.	4 (25%)
3) External coach		
a) Added value of coaching ^b^		
Strongly	*7* (47%)	*6* (38%)
To a reasonable extent	*6* (40%)	*9* (56%)
To some extent	*2* (13%)	*1* (6%)
b) Coaching necessity for (continued) implementation		
Yes	*5* (33%)	*9* (56%)
No	*7* (47%)	*4* (25%)
I don’t know	*3* (20%)	*3* (19%)
c) Competence of coach ^c^ perceived by project leader		
Competent or very competent	*11* (73%)	N.A.
Not competent/not incompetent	*1* (7%)	N.A.
Other ^d^	3 (20%)	N.A.

^*a*^ *N* = 6 coaches for *N* = 16 nursing homes

^*b*^ Scale: Not at all/to some extent/to a reasonable extent/strongly

^*c*^ Likert Scale: Very incompetent/incompetent/not competent-not incompetent/competent/very competent

^d^ Differences between competence in content and process, such as incompetent on content and competent on process

The problem analysis was often perceived to create urgency for change because researchers provided MPTs with information about inappropriate PDU and NPS management. This initiated a dialog and resulted in NHs comparing themselves to other organizations. The fixed measures motived NHs to achieve change, and the provision of interim results not only provided valuable insights into their progression but also provided an opportunity to make changes. Although the ability to tailor action and implementation to each organization was evaluated positively, the process was considered both time consuming and complex.

2) Internal project leader and MPT

The role of the internal project leader was considered essential, but this was highly dependent on their skills, such as creating support, engagement, and informing staff. The MPT was also considered relevant because of the multidisciplinary nature of managing NPS and inappropriate PDU. Actively involving the MPT in formulating the problems and solutions was positively evaluated:

*Internal project leader:* “The actions are self-created, which creates greater support. You can impose all sorts of things but that won't work. It really has to come from themselves, what they think might work.”

3) External coach

External coaching was considered especially useful for translating the problem analysis to a tailored AIP, with many stating that this process was difficult. However, although many respondents thought that external coaching was necessary, others considered it relevant but non-essential. A positive appraisal is illustrated by the following quote:

*External coach:* “They had project groups and those people were quite driven to get started. They definitely had a clear direction... and they acted on this as well.”

### Intervention quality: extent of performance

Results for the RID intervention’s extent of performance are depicted in Table 4.

1. Organizing efforts of stakeholders
  *Researchers:* As intended, the researchers carried out a kick-off meeting in each NH.
  *MPTs:* All 16 NHs formed MPTs from various disciplines, and in most cases, attendance was good. Several other NHs (no. 2, 3, 5, 6, 8, 12) had a low attendance level (Table 4, column 1a, and Additional File 1).
  *External coaches:* The number of meetings (Table 4, columns 1b and 1c) with the external coach and the MPT on location varied substantially between NHs (range 5–13), as did the number of (phone) meetings between the external coach and internal project leader (range 0–12).
2. Problem analysis

**Table 4:** Extent of Performance of the RID intervention

NH	1a) Atten-dance MPT	1b) Meetings coach + MPT	1c) (Phone)-meetings coach + PL	3a) AIP ^*^ created	3b) Contribution coach, PL, MPT in designing AIP	3c) Adjustments AIP based on feedback	4a) Start with implementation in weeks	4b) Execution ^A^ actions of AIP as intended	7) Adjustments AIP based on interim results
1	76–100%	9	0	Yes	(Very) large	Yes	Within 8	8.4	Not necessary
2	0–25%	7	5	Yes	(Very) large	Yes	Within 8–16	8.5	Yes
3	26–50%	13	12	Yes	(Very) large	Yes	> 16	5.4	No
4	76–100%	7	3	Yes	(Very) large	Yes	Within 8	7.0	Yes
5	26–50%	9	7	Yes	(Very) large	Yes	Within 8–16	6.4	Yes
6	26–50%	5	4	Yes	(Very) large	Yes	Within 8–16	7.9	Not necessary
7	51–75%	8	3	Yes	(Very) large	Yes	Within 8–16	7.9	Yes
8	26–50%	11	12	Yes	(Very) large	Yes	Within 8–16	6.3	Yes
9	76–100%	9	8	Yes	(Very) large	Yes	Within 8–16	7.0	N.A. ^B^
10	51–75%	6	2	Yes	(Very) large	Yes	Within 8–16	3.4	N.A.
11	76–100%	5	3	No.	N.A.	N.A.	> 16	5.0	N.A.
12	26–50%	7	2	Yes	(Very) large	Yes	Within 8–16	6.8	N.A.
13	76–100%	8	4	Yes	(Very) large	Yes	Within 8	7.3	N.A.
14	76–100%	5	7	Yes	(Very) large	Yes	Within 8–16	7.8	N.A.
15	76–100%	5	1	Yes	Reasonable	No	Within 8–16	5.3	N.A.
16	76–100%	7	10	Yes	(Very) large	Yes	Within 8–16	6.3	N.A.

*Abbreviations*: *AIP* (tailored) Action and Implementation Plan, *MPT* Multidisciplinary Project Team, *NH* Nursing Home, *PL* (internal) Project Leader

^A^ Mean of implementation scores of each action from AIP: 10-point scale (0 not at all implemented as intended – 10 totally implemented as intended) per action

^B^ Not Applicable: nursing homes 9–16 were in the deferred control group (start with the intervention in the second 8-month period and had therefore no interim results)

The researchers carried out a problem analysis at each NH and presented the results to all MPTs.

3) Designing tailored AIP

In NH 11, communication issues between the person who decided to participate in the project and the persons responsible for executing it delayed the process to the extent that no AIP was created (Table 4, column 3a). The AIPs otherwise contained the following actions: multidisciplinary and methodical working (including use of person-centered interventions), education and training, and adaptations to the living environment. Generally, actions in the AIPs addressed the identified problems. Apart from NH 11, which lacked an AIP, all NHs were given feedback on their AIP by the external coaches and researchers. The contributions of the external coach, internal project leader, and MPT in designing the AIP were large (Table 4, column 3b). Also, apart from NH 11, all NHs adjusted their AIP based on the feedback given by the external coach and researchers (Table 4, section 3c).

4) Implementation of tailored AIP

Only NHs 1, 4, and 13 did not need more than the allocated 8 weeks to start AIP implementation, and NHs 3 and 11 only started implementation after 16 weeks (Table 4, column 4a). The mean implementation scores for executing the AIPs on a 10-point scale (range, 3.4–8.5) were below 6.0 in NHs 3, 10, 11, and 15 (Table 4, column 4b).

5) Monitoring progression

The researchers carried out an interim measurements as intended, and the relevant MPTs were given interim results at 8 months.

6) Stimulating progression

All external coaches discussed the interim results within each MPT.

7) Adjustments to tailored AIP

Most NHs adjusted their AIP after discussing the interim results. Changes mostly focused on not only what was important to keep doing or what was likely to succeed but also defining actions and strategies more precisely. Two NHs (no. 1, 6) had positive results and decided to continue with the original AIP, and one NH (no. 3) did not change the AIP despite negative interim results. In the latter case, the MPT argued that further change was unwise because they had barely started to implement the original AIP (Table 4, column 7).

8) Providing final results

The researchers carried out a final measurement at each NH and presented all MPTs with the final results.

### Barriers and facilitators to implementation

The barriers and facilitators identified from the interviews could be categorized into three of the CFIR themes: intervention characteristics, inner setting, and process [37]. Description of the CFIR topics are given in Additional File 2.

#### Intervention characteristics

Involving NH staff in addressing the problems, needs, and solutions created the engagement and support needed for implementation, but involving so many people also seemed to slow the process. This was key to why some perceived the planned implementation period as too short. Several NHs in the deferred intervention group indicated that the amount of time between project registration and action was too long, which reduced their enthusiasm. It was also perceived that NH staff sometimes struggled to translate knowledge into practice after education or training, but on-the-job coaching was considered to be helpful in applying what was learned into practice.

#### Inner setting

Several barriers were common, such as reorganizations, staff shortages and turnover, and communication issues within and between disciplines (i.e., too little contact, criticizing each other, or not being receptive to feedback). Another perceived barrier was the use of self-directed teams that had responsibilities and duties assigned to teams without a formal lead. Some NHs embraced change whereas others seemed more reluctant; for example, it was observed that some MPT members questioned every suggestion or assumed that colleagues would not keep to the agreements made. Limited self-reflection was also mentioned, with respondents indicating that MPT members and NH staff sometimes found it hard to accept that the level of PDU in their NH was high, despite feedback and evidence to the contrary. Time pressure interfered with implementation in other instances, and in some cases, the NH management did not grant their staff the time needed to complete the project; however, several respondents indicated that the issue of time constraints, whether perceived or real, was about setting priorities. Implementation was facilitated when NHs developed a view or vision on PDU with sufficient alternatives, because it allowed them to build on a basic level and focus on finetuning agreements. Levels of innovative power were different among NHs, and it appeared that those used to participating in research had an easier time with implementation. It was also easier to implement the RID intervention when it could be integrated with other projects, but this was at the cost of the multiple projects placing excess demands on staff.

#### Process

The involvement of stakeholders, such as internal project leaders, physicians, and psychologists, differed among the NHs, but if these key persons could continue their efforts, staff turnover did not negatively impact the project. Notably, arranging for proper transition facilitated implementation when there was turnover in these positions. Active participation by management also conveyed to the MPT that the project was important. In some cases, it also seemed that the internal project leaders lack the skills and personality for their role. In fact, a change in the internal project leader was not a barrier when that person was replaced by someone who was better suited to the role.

*External coach:* “She read all information about the project, was well prepared, and brought structure; she worked according to a fixed agenda, with notes and action lists. She asked about intrinsic motivation (why are you in the project team) and held them accountable … She was decisive and sought connection with relevant parties such as the policy advisor and manager.”

External coaches mentioned that there were variations between NHs, requiring that they customized their approaches to each NH. Often external coaches were perceived to facilitate implementation by providing structure and reflection, and in some instances, they “scaled up,” examined the internal dynamics, and tried to create engagement by addressing the need for change and the relative advantage. Nevertheless, staff in some NHs were perceived to remain reluctant despite these strategies. An issue was that the expectations of the project were not always in line with what was communicated. It was sometimes expected (mostly by internal project leaders, but also by a few external coaches) that the results of the problem analysis would yield manageable and directly applicable information, without the need for reflection and translation by the MPT and external coach (e.g., “what does this information mean?” and “what do we want to do?”). Also, some MPT members failed to engage in the project either because the external coach was treated as the main carrier of the project or because MPTs were not open to being coached.

*External coach:* “I can be very facilitating, and I can be a guide, but the organization must act itself. I can't tell people what to do ... I can only advise ‘it is smart to do this’ or ‘you can choose from this and this and choose for yourself what fits your organization’ best.”

Although MPT members were generally very enthusiastic, this did not guarantee results because the ability to move forward was sometimes perceived as limited. According to respondents, overestimating the ease of implementing the innovations led some NHs to create AIPs that included either non-specific or excessive actions that could result in an unclear division of roles and responsibilities. Despite researcher feedback in which these concerns were stressed, no changes were made by the relevant MPTs.

## Discussion

In general, the RID intervention with PAR, external coaching, and tailored information provision was evaluated positively. The local problem analysis, coupled with the presence of external coaches and researchers, often generated an impetus for action. However, the overall process was complex and time consuming from the problem analysis to the design of a tailored AIP, with external coaching being key during this transition. Coaching also seemed to have an empowering effect, with some NHs even considering it a prerequisite for ongoing implementation. Nevertheless, important issues were that the set time period of 8 weeks for problem analysis and AIP design was too short and that the extent of performance was suboptimal, with several differences emerging among the NHs. For example, 4 of the 16 NHs had short implementation periods because of delays. This might have resulted in limited execution of key actions that may have reduced the effectiveness of the RID intervention in these NHs.

Actively involving NH staff throughout the implementation process was deemed essential by respondents. This is consistent with existing literature showing that bottom-up approaches that include local stakeholders are key to gaining support for, and the adoption of, a given intervention [38]. A potential downside of PAR is that actively involving staff in the whole process meant that it was often impossible to complete implementation within 6 months, because in practice, the process can be very time intensive. Staff turnover and reorganizations further complicated implementation, which is again consistent with other findings [24, 25]. To date, these contextual/environmental characteristics have been viewed as confounders or barriers, but it might be more appropriate to accept them as normal conditions into which interventions must be integrated [39]. In our view, placing preconditions on organizations before they can participate (e.g., requiring stability) is unrealistic, and we must instead better account for discontinuity due to staff turnover or reorganization during implementation processes. As such, because the issue of staff turnover cannot be resolved, the challenge lies in learning to implement a new strategy in a changing context. Our results suggest that the negative impact of such change can be mitigated if communication and takeover are handled well.

The extent to which NH staff are willing and able to implement nonpharmacological strategies is important, considering that many nonpharmacological strategies depend upon implementation by NH staff [40]. Earlier research has concluded that the readiness of staff for change must be considered during implementation planning [41]. Similarly, we found that differences existed in the extent to which NH staff were open to change. For example, time pressures often were used as an argument for impeded implementation. Although this common barrier has been mentioned in other studies [42–44], some of our external coaches noted that time pressures were often an issue of perception and stressed that the true problem was about setting priorities. Although literature illustrates that dealing with NPS is indeed a priority in long term care, taking into account that NPS can result in distress amongst nursing staff [45–47], the uptake of nonpharmacological treatments in daily practice is still limited. Nonetheless, the results of our study indicate that despite discussing the relevance of change (i.e., the degree of inappropriate PDU in the NH) and the added value of intervening, some NHs remained reluctant to change. In these instances, creating engagement based on content and reflection with an external coach could therefore have been ineffective. Nevertheless, assessing and supporting an organization’s readiness for change might facilitate successful implementation. This is in line with Pimental et al. stressing that an organization’s readiness for change is essential and is a function of organizational members’ shared commitment to implementing change and a shared belief in their collective capability to do so [48].

Communication issues impeded implementation in our study, which is again consistent with previous research [24, 26]. Notably, our problem analysis revealed that many NHs struggled with there being little interdisciplinary contact. Although staff in some NHs recognized this as a point for improvement, budget cuts meant that NH management were often unwilling to invest in the AIP. Also, some MPTs had expectations of the external coaches and researchers that were too high, underlining a need to communicate what they can expect more clearly and to empower teams to feel confident in taking action themselves.

We confirmed two findings of our recently published systematic review on the barriers and facilitators of complex interventions for residents with dementia in long-term care [49]. First, limited skills of internal project leaders impeded implementation in some cases. As suggested in our earlier review, greater care may be needed to ensure that we select competent and suitable staff to drive change (e.g., identifying a role model whose advice is accepted by colleagues). Second, as concluded in our review, we found that nursing staff occasionally struggled to apply their newly acquired knowledge in practice, indicating that the education or training methods we adopted may not have been suited to their learning styles. This also underlines the importance of interventions that are compatible with the intended users. Approaches such as on-the-job coaching let to enhanced applicability and should be considered in the future.

Finally, despite the aim of this study, no role was defined for pharmacists. In the Netherlands, it is common for organizations to have monthly pharmacotherapeutic consultations and annual mandatory medication reviews with their supervising pharmacists [50]. If organizations wanted additional involvement from their pharmacist, this could be included in the tailored AIP. Nevertheless, in retrospect we do argue that informing pharmacists about the study and the possibility to be involved could have been of added value.

In a subsequent study, it could also be interesting to include the impact of NPS for nursing staff and relate this to compliance. In addition, qualitative data indicates that improvements were perceived, for example related to multidisciplinary collaboration. This was not measured as an outcome. Taking this into account, and considering that many of the implemented actions relate indirectly to PDU, we stress that future trials emphasize a broader range of outcomes such as knowledge, multidisciplinary collaboration, or use of person centred interventions.

Given that residents and their family members are relevant stakeholders and can be an important motivator to change, future studies should consider an implementation strategy in which they can contribute to realizing change.

## Strengths and limitations

The use of a mixed methods approach ensured that we obtained an extensive and thorough insight into all aspects of our process evaluation. However, a critical comment is appropriate regarding our respondents. The external coach and internal project leader represented our respondents, while many other stakeholders participated in the study as well. Considering this, we have included a relatively small number of respondents. Moreover, various stakeholders have different professions and areas of expertise, which is likely to influence somebody’s vision, opinion and, as a result, evaluation of the process. Consequently, the data reflects the opinion of these two roles and might be a limited representation of reality. Nevertheless, both roles were key to the RID intervention and we considered the majority of the respondents to be relatively well aware of the perceptions of others involved in the process. Due to turnover, some respondents were not involved from the beginning of the intervention, which possibly affected completeness of the data. The evaluation parameters we used may represent an important limitation given the tailored nature of our intervention. It was overly complicated to determine what parameters needed to be included and how these should be operationalized to evaluate the implementation. For instance, the use of a mean implementation score for AIP actions was deemed suboptimal because each score does not acknowledge the different importance of each action. Finally, recall bias may have influenced the results because the data were only collected after the project, and this may have influenced the reliability and comprehensiveness of our results given the turnover of external coaches and/or internal project leaders in some NHs.

It should be noted that we deviated from our study design in two aspects [31]. First, although the tailored information provision and external coaching were described as implementation strategies, we now believe they should be considered inherent to the core intervention. This is consistent with many complex interventions that incorporate implementation strategies in this way [49]. Given that the process evaluation model we used assumes that intervention and implementation strategies be separate [34], this model may need to be adapted for process evaluations of complex interventions. The second deviation from our design concerns the examination of barriers and facilitators. To ensure that this aspect was well examined, we developed questionnaires based on common factors reported in other studies [24, 26, 34, 51] and detailed in the CFIR [37]. However, it was difficult to answer the questions without ambiguity, which meant that the interviews were more useful for in-depth exploration.

## Conclusions

We hypothesized that implementation would be facilitated using an intervention with PAR, external coaching and tailored information provision. Although these elements were appreciated and implementation may indeed have been facilitated, the added value and effectiveness of these elements depends on a large number of factors. Consequently, the level of implementation (e.g. extent of performance) differed between NHs.

The RID intervention was evaluated positively, but it was also considered to be time consuming and complex. Although external coaching was certainly considered relevant, it was not considered indispensable, with its added value rated differently across organizations. That said, the external coaches stimulated implementation and even had a role in mitigating the effects of some of the barriers we encountered (e.g., facilitating proper takeover of key roles). Also, the effectiveness of coaching may have been dependent on a range of factors, including the organization, openness to coaching and change in general, and whether NH staff and management can be motivated by arguments, facts, and numbers. Staff turnover and reorganization were recurring themes in the analysis of barriers, and given that these are ubiquitous to normal practice, we believe that any future implementation strategy should address innovating within the broader confines of an ever changing environment. Despite our efforts, we partially encountered well-known barriers. This process evaluation provides insights into the implementation of a complex intervention, but it also shows how difficult it is to realize quality improvement and culture change within NHs. This takes time and affects all different kinds of stakeholders and organizational levels. Therefore, future studies do well to assess and support organizations in their readiness to change. Given that the extent of performance of the NHs varied, sensitivity analyses are appropriate when investigating the effects of the RID intervention, taking into account the week in which nursing homes started with implementation and the degree to which actions were implemented as intended. We stress that future trials emphasize a broader range of outcomes such as knowledge, multidisciplinary collaboration, or use of person centred interventions.

## Supplementary Information

**Additional file 1.**

**Additional file 2.**

## Data Availability

The datasets generated and/or analyzed during the current study are not publicly available considering all data is in Dutch but are available from the corresponding author on reasonable request.

## Abbreviations

AIP: Action and Implementation Plan
CFIR: Consolidated Framework for Implementation Research
CONSORT: Consolidated Standards of Reporting Trials
DSCUs: Dementia Special Care Units
MPT: Multidisciplinary Project Team
NPS: Neuropsychiatric Symptoms
NH: Nursing Home
PAR: Participatory Action Research
PDU: Psychotropic Drug Use
PDs: Psychotropic drugs
RCT: Randomized Controlled Trial
RID: Reducing Inappropriate psychotropic Drug use
SPSS: Statistical Package for the Social Sciences
UMC: University Medical Center
