# Supplementary material for: Process evaluation of a tailored intervention to Reduce Inappropriate psychotropic Drug use in nursing home residents with dementia
Source: BMC Geriatr. 2021 Jul 3;21:414. doi: 10.1186/s12877-021-02357-w (PMC8254904; doi:10.1186/s12877-021-02357-w)
Supplement: Supplementary file 1 — Additional file 1. [file 12877_2021_2357_MOESM1_ESM.docx]

**Additional File 1.** Attendance Multidisciplinary Project Team

| Nursing home | Physicians | Psychologists | Nurses |
| --- | --- | --- | --- |
| 1 | 76%–100% | 51%–75% | 76%–100% |
| 2 | 0%–25% | 0%–25% | 76%–100% |
| 3 | 0%–25% | 26%–50% | 51%–75% |
| 4 | 26%–50% | 76%–100% | 76%–100% |
| 5 | 0%–25% | 26%–50% | 26%–50% |
| 6 | 26%–50% | 26%–50% | 26%–50% |
| 7 | 51%–75% | 76%–100% | 51%–75% |
| 8 | 26%–50% | 26%–50% | 76%–100% |
| 9 | 51%–75% | 76%–100% | 76%–100% |
| 10 | 76%–100% | 51%–75% | 26%–50% |
| 11 | 76%–100% | 76%–100% | 76%–100% |
| 12 | 51%–75% | 26%–50% | 0%–25% |
| 13 | 76%–100% | 76%–100% | 76%–100% |
| 14 | 76%–100% | 76%–100% | 76%–100% |
| 15 | 76%–100% | 76%–100% | 76%–100% |
| 16 | 76%–100% | 76%–100% | 26%–50% |

- In case of differences in answers between coach and internal project leader, the most conservative (low) score was depicted, given the importance of distinguishing nursing homes that have implemented less successfully.

- 4-point scale: 0%–25%/26%–50%/51%–75%/76%–100%.
